# Comprehensive Review of Epidemiology and Treatment of Snakebite Envenomation in West Africa: Case of Benin

**DOI:** 10.1155/2024/8357312

**Published:** 2024-04-08

**Authors:** Ayékotchami Jacques Dossou, Adandé Belarmain Fandohan, Timothy Omara, Jean-Philippe Chippaux

**Affiliations:** ^1^Research Unit in Forestry and Conservation of Bioresources, National University of Agriculture, School of Tropical Forestry, BP 43, Kétou, Benin; ^2^Department of Chemistry, College of Natural Sciences, Makerere University, P.O. Box 7062, Kampala, Uganda; ^3^Paris Cité University, Research Institute for Development, MERIT, Paris F‐75006, France

## Abstract

Snakebite envenomation (SBE) constitutes a public health, social, and economic problem affecting poor communities in intertropical and subtropical regions. This review sought to synthesize literature on snakebite envenomation in Benin to highlight research perspectives and strategies for better management of the menace. A literature search performed in multidisciplinary electronic databases showed that the prevalence of SBE is high in Benin, but the incidences, associated morbidities, and mortalities are greatly underestimated. Most snake envenomations are by *Echis ocellatus* in Northern Benin during the rainy season. Adults involved in agricultural activities are the most affected. The absence of antivenin serum in the most affected areas explains the preference for alternative and traditional medicine as the first-line treatment for SBE in Benin. In conclusion, it would be imperative to revitalize the snakebite reporting system in order to have better epidemiological data and to develop a sustainable national strategy for the control and management of snakebite envenomation.

## 1. Introduction

People living in intertropical and subtropical areas around the world (Africa, Asia, and Latin America) are vulnerable to snakebite envenomation (SBE). Globally, at least 5.4 million people are bitten by snakes each year. Of these victims, 81410 to 137880 die, and around three times as many amputations and other permanent disabilities result [1]. Asia takes the highest burden of SBE (up to 2 million people envenomed by snakes each year), whereas Africa accounts for about 435,000 to 580,000 (20%) of the total global cases [1].

SBE is ranked among the major public health problems in Africa, especially in West Africa, where more than 95% of cases occur among women, children, and farmers in rural areas [2, 3]. SBE causes haemorrhage, tissue necrosis, and/or renal failure, limiting the productive potential of the victims [2, 4, 5]. More than 314,000 cases of snakebites, 7,300 deaths, and nearly 6,000 amputations occur in sub-Saharan Africa each year [2]. Halilu et al. [5] estimated the annual burden of SBE to be 1.03 (0.80–1.28) million disability-adjusted life years, which is higher than the burden of many NTDs. Most of the SBEs are reported in areas characterized by minimal access to health facilities and antivenom sera, poor road transport, agrarian-based livelihoods, and chronic poverty [3–5]. Although the incidences, morbidities, and mortalities from SBE are highly underestimated, the menace is gaining more attention and was re-acknowledged in 2017 by the World Health Organization as one of the neglected tropical diseases (NTDs) [4, 6].

According to the GBD 2019 Snakebite Envenomation Collaborators, the menace of SBE is an important cause of preventable deaths, and the WHO set a goal to halve snakebite mortality by 2030 [7]. Due to the inadequate health statistics and epidemiological data in most health monitoring facilities, it is difficult to assess the real extent of the public health issues caused by SBE in Africa [7]. As such, the foregoing statistics likely hold important bias and little information on West African countries such as Benin. Unlike the case with other NTDs that are practically eradicable, SBE cannot be completely eliminated because some of the venomous snake species are key players in complex ecosystems such as the biological control of pests such as rodents [7]. Aside from this knowledge, there is still a continuous need to effectively control the burden of SBE to minimize its physical, psychological, and socioeconomic impacts. This could be realized through the compilation of statistics on the incidence, intensified management coupled with adequate investment in new diagnostics, treatment, and prevention tools, as well as ensuring equitable access to affordable and effective antivenoms [7].

The objective of this study was to review the literature on the epidemiology (incidences, mortalities, seasonality, responsible snake species, and severity assessed by case fatality rate) of SBE in Benin, West Africa. The available treatment options as per the encountered literature were also discussed.

## 2. Methodology

This review is a synthesis of the literature on SBE in Benin, a country in West Africa, focusing on incidence, mortality, seasonality, responsible species, and severity; demographic data of victims (age, sex, and occupation); availability of the antivenom; the therapeutic course of the victims; and the plants used in the management of SBE. Publications were obtained from online databases, namely, AGORA, Scopus, PubMed, ScienceDirect, Google Scholar, and Google, using keyword combination as follows: Benin AND snake OR ophidian, Benin AND envenomation OR antivenom OR antivenin, Benin AND epidemiology, snakes, snakebite AND Benin.

All published documents, articles, conference proceedings, and book chapters published up to January 2023 were considered. To refine our research, we also took into account grey literature. It consisted of collecting all the theses and dissertations related to SBE in Benin. The general characteristics of included publications are summarized in Table 1. Overall, 26 papers, 9 theses, and 1 dissertation addressing SBE were found.

## 3. Results and Discussion

### 3.1. Evolutionary Trend of Scientific Publications on SBE in Benin

**Figure 1:**
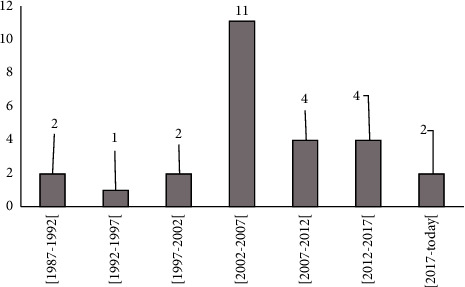
Evolution of the annual number of scientific publications on SBE in Benin (1987–present).

The first attempt to gather data on SBE in Benin dates back to 1987 and 1988. Fayomi et al. [8] and Chippaux [9] first acknowledged the need for reliable data on epidemiology and the incidences of SBE in Benin in order to better plan and implement an SBE management policy or strategy. Between 2002 and 2007, the topic gained research interest with 11 papers published. After 2007, however, interest in the subject considerably decreased and only 10 papers were published over the 13 years (Figure 1). Later, 9 theses and 1 dissertation were devoted to the problem of SBE in Benin focusing on various aspects. These theses were conducted between 2005 and 2020, with 3 theses between 2005 and 2010; 2 theses and a dissertation between 2010 and 2015; and 4 theses between 2015 and 2020. They were concerned with the epidemiological, clinical, and therapeutic aspects of SBE, efficacy, and tolerance of Inoserp® Pan-Africa, complications, acute kidney disease following severe envenomation, and the role of ultrasound in SBE treatment. It would be expected that the reconsideration of SBE as a neglected tropical disease by the WHO in 2017 could have driven more research interest in it between 2017 and 2020.

### 3.2. Incidences and Lethality of Snakebite Envenomation in Benin

#### 3.2.1. Retrospective Studies

Retrospective studies have allowed the acquisition of a posteriori data on the epidemiology of SBE in Benin. The study conducted by Fayomi et al. [10] on SBE from 1993 to 1995 recorded an average annual incidence of 3,138 cases with 1.5% case fatality rate (i.e., 142 deaths out of 9,414 registered cases). Variation and evolution in a number of victims were observed during this period. From 1994 to 1999, an incidence of more than 4,300 cases was noted each year [11]. The number of cases recorded in this period varied from 4,146 to 4,685. Beyond the significant variation of statistics from these two relatively close periods, it is necessary to highlight that there were some statistical inconsistencies concerning the data reported. As such, while Fayomi et al. [10] indicated 2,963 and 3,853 SBE incidences in 1994 and 1995, a subsequent report by the same research group mentioned 4,146 and 4,141, respectively, for the same years.

It is therefore obvious that these variations reflect inconsistencies during data collection, even though the data were collected from the same management system as the Ministry of Health. The observed discrepancies could be due to missing information due to loss of records, absence of health personnel, and poor monitoring facilities [11]. To reduce these inconsistencies and variations, prospective studies that provide more precise and reliable information and data, particularly in the case of SBE, could be recommended [2].

**Figure 2:**
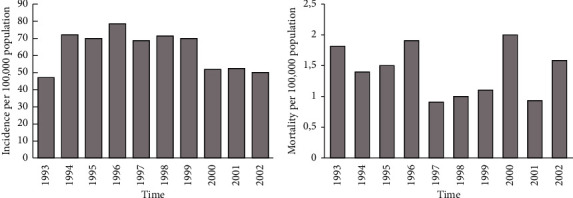
(a) Annual incidence of SBE (per 100,000 populations) in Benin from 1993 to 2002; (b) annual mortality of SBE (per 100,000 population) in Benin from 1993 to 2002.

The average incidence related to SBE recorded from 2000 to 2003 across Benin was 3,651 cases, with a fatality rate of 1.57 from 2002 to 2003 [12]. From 1993 to 2002, the incidence averaged 63.8 cases per 100,000 population with 1.41 (±0.13) per 100,000 population mortality [10–12]. Rather, Chippaux [13, 14] estimated records of SBE at an average of 4,500 records with almost 15% case fatality rate each year (Figures 2(a) and 2(b)).

Some retrospective studies have been conducted in different areas of Benin and have shown a more or less significant increase in SBE cases and mortalities (Table 2). The incidences of SBE were high in these reports, although these data only take into account information from public health facilities, without accounting for private health centres and cases treated with traditional medicine, especially when we know Beninese for herbal remedies. It is also evident that there is a significant underestimation of official figures for the incidence and mortality of SBE in Benin. The retrospective nature of these studies does not allow taking into account all the information related to bites, which is likely to contribute to better management of care. This limit could well be lifted by prospective studies, which provide reliable and up-to-date information useful for the prevention and management of SBE. Beyond the data obtained through this study and the importance of retrospective studies, the absence of a SBE data collection platform constitutes a fundamental limitation for obtaining reliable data. Indeed, the loss of certain data registers or their deterioration constitutes a weakness of the retrospective data collection methodology within the framework of this study. It would therefore be important to improve the system of declarations and the collection of epidemiological data on SBE in order to know in real time the situation relating to it for better care and consideration in the integration of the country's health policy.

#### 3.2.2. Prospective Studies

A study conducted by Massougbodji et al. [23] in Benin denoted up to 486 SBE with 22% deaths over 12 months. Mortality from SBE was significantly greater in rural areas than in urbanized areas, probably due to poor health systems, inadequate equipment in rural health centres, and untrained medical staff with regard to SBE handling. This prospective study also enabled access to epidemiological and socioeconomic data such as sex, age, profession of the snakebite victims, presentation time, clinical symptomatology, bite season, treatment, and costs. These data often are rarely available in retrospective studies. Moreover, prospective surveys performed in 7 villages in Benin showed that 17 cases of SBE only had one death (case fatality rate of 5.9%) in a population of 1,300 inhabitants [13]. Finally, an Antivipmyn® Africa efficacy trial carried out in 289 SBE cases resulted in 9 deaths due to late presentation [24]. This trial re-emphasized the need for antivenin sera to be well stocked in public health facilities to reduce mortalities and morbidities due to SBE in Benin.

In addition, the few prospective studies carried out on an ad hoc basis in Benin were conducted in the northern part of the country (Table 3), which emphasizes the gravity of SBE in this part of the country. Prospective studies benefit from robustness but are still poorly performed because of limited time and resources. Nonetheless, more prospective studies are recommended to understand the characteristics of SBE and establish policies and strategies for its prevention and management. It should be noted that the prospective studies on SBE in Benin are not always in the strict sense of the theme. The methodology for carrying out these studies, in particular the relatively short time considered and the very imprecise objectives, constitutes limits for obtaining robust data on SBE. Indeed, prospective studies are essential because they make it possible to follow groups of subjects over a period, identify risk factors, evaluate the effectiveness of interventions, and establish cause-and-effect links. These studies provide a solid foundation, thereby contributing to the prevention of diseases, the improvement of health care, and the advancement of medical and scientific knowledge.

#### 3.2.3. Household Surveys

Household surveys have specific objectives and make it possible to collect cross-sectional and qualitative information from populations and victims related to SBE [28]. The household survey carried out by Chippaux [13] in 13 villages in Benin collected data on the evolution of SBE and symptomatology. Similarly, the household survey conducted in Pobè assessed the attitudes of the population that could facilitate SBE understanding and management [19]. Combining household surveys with prospective studies could make it possible to determine, apart from relevant and real epidemiological data and statistics, the sociocultural, economic, and geophysical factors influencing the perception of communities at risk of SBE and the use of treatment. These data will make it possible to modify behaviour, policies, and practices for better management of SBE in Benin.

#### 3.2.4. Snakes Responsible for SBE in Benin

The ophidian fauna of Benin is not fully known. However, it should be noted that SBE in Africa is caused by cobras, mambas, and vipers from the Elapidae and Viperidae families [29]. Most studies in Benin have shown that vipers (e.g., *Echis ocellatus* Stemmler, *Echis leucogaster* Roman, *Bitis arietans* Merrem, and *Causus maculatus* Hallowell) are responsible for more than 90% of the SBE cases [17, 18, 22, 25, 27]. Small vipers (*Echis* and *Causus* species), large vipers (*Bitis* species), and *Naja* species are reported to be responsible for most SBE in Benin [10]. In particular, *Echis ocellatus* was reported to be the major responsible for SBE in Northern Benin [13]. Its venom was detected in the blood of 82% of patients treated by Antivipmyn® Africa in Benin [24].

### 3.3. Epidemiological Data on SBE in Benin

#### 3.3.1. Geographical Distribution of SBE in Benin

Numerous studies have shown that the incidence and severity of SBE are unevenly distributed in Benin. The incidences and especially mortalities showed a positive correlation with latitude [10, 11, 13, 23]. Thus, the northern part of the country clearly shows higher incidences of SBE. According to Fayomi et al. [10], the bimodal climate and the rugged relief prominent in the North provide optimum niches for venomous snakes in Africa, especially vipers and cobras. This geographic gradient could be justified by two main factors. However, the ophidian population may be limited by strong demographic pressure and urbanization linked to the density of the human population and their activities. As a result, Northern Benin is much less urbanized and has a lower population density than Southern and Central Benin. The environmental conditions (sunshine, relative humidity, temperature, etc.) influence the behaviour and growth of snakes. It has been established, for example, that an increase in temperature affects the distribution of snakes, potentially increasing the risk of SBE [30].

#### 3.3.2. Circumstances of SBE

SBE results from an unexpected natural encounter between two protagonists (humans and snakes), which may be due to the convergence of activities. In Benin, most cases of SBE occur in the field, during agricultural activities (adults) and/or recreational activities (and complementary food seeking in younger victims). At least 75% of the bites are the direct consequences of agricultural activities and barefoot movements related to these activities and hunting [10, 14, 19]. The use of rudimentary agricultural techniques is one of the main risk factors for the high burden of SBE among farmers [14]. Modernization of agriculture could therefore considerably reduce the risks of SBE, especially in rural areas. As a preventive measure among farmers, protective attires (boots, long pants, and gloves) must be worn.

#### 3.3.3. Age and Gender

Victims of SBE in Benin are mainly young adults. Almost 75% of men over the age of 15 suffer from bites at least once [10, 14, 19]. Women are twice less affected than men [14]. This could be due to differences in agricultural activities between men and women (land preparation, clearing, and ploughing are more often performed by men, while women and children take care of sowing, weeding, and harvesting), or cultivation of different plant varieties (e.g., cash crops for men versus food crops for women). However, it could result in gender-specific risks linked to contact with distinct species, either because of the seasonal variation of snake species or because each type of plantation influences the population composition and demographics, including the density of snakes [31, 32]. Consequently, young men are more exposed than children, women, and the elderly. However, SBE in children represents 33% of total cases [10] because children represent nearly half of the overall Benin population. Bites occur mainly in the lower limb, especially below the knees [14, 15].

#### 3.3.4. Seasons

Season (or climate) is also reported to highly influence the number of SBEs. The incidence of SBE is linked to agricultural activities, which frequently put humans in contact with the natural environment and therefore to the behaviour of snakes and the agricultural calendar. Rainfall is a determining factor in human and ophidian behaviour [14]. Most studies in Benin and several countries of the subregion (Senegal, Niger, Mali, Nigeria, and Cote d'Ivoire) have shown that the majority of SBE takes place during the rainy season [11, 19]. Massougbodji et al. [23], however, mentioned that the highest incidences of SBE occurred during the dry season. This could be explained by hunting, land preparation activities for the next agricultural season, and recreational activities of the younger victims. High heat during this period forces the reptiles to seek cooler shelters, which often happen to be areas of human activities. A gradual increase in cases is observed each year from March to August, which is the period of intense agricultural activities in Benin [10, 15, 16]. Also, in the savanna zone, the reproduction of snakes (couplings and laying) occurs mostly during the rainy season, which corresponds to human activities.

### 3.4. Serious Cases of SBE in Benin

About half of the bites show no signs of envenomation [2]. These are asymptomatic bites by either nonvenomous or venomous snakes that do not inject venom (dry bites). According to Massougbodji et al. [23], 28% of the victims have oedematous and cardiovascular syndromes (shock), 20% showed haemorrhagic syndrome, and 10% experienced respiratory distress. Chippaux [14] estimated that 66% of victims presented with inflammatory syndromes, whereas only 12% and 5% had haemorrhagic syndromes and necrosis, respectively. Furthermore, the severity of SBE is favoured by factors such as the following.

#### 3.4.1. Consultation Period

Delay in consultation is a determining factor in the severity of SBE [23]. Most studies conducted in Benin have shown that consultations were late in health facilities with an average delay of 48 to 72 hours. Djomga [33] found an average consultation time of 41 hours with extremes ranging from 6 hours to 96 hours. The admission time ranged from less than an hour to 7 days [27]. However, Kouomboua Mfin [34] found that the majority of victims consulted 7 days after SBE. Inadequate access to medical care for patients living on the outskirts of urban areas, lack of awareness of the severity of the disease, high cost of hospital treatment, and traditional first-line treatment may explain delays in consultation.

#### 3.4.2. Prehospital Care

Most patients received traditional treatment before admission. This treatment is often made of incisions, scarifications, use of black stone, and medicinal plants [17, 18, 22, 25, 27]. The almost systematic recourse to this treatment, which could be detrimental to victims, would be due to the perception of communities of SBE as being a divine punishment or a spell and the high cost of the hospital treatment. Totin [26] found that the average cost of hospital care amounted to 170,270 FCFA (259.96 Euro) with extremes of 15,000 (22.90 Euro) and 401,750 FCFA (613.36 Euro) at the Borgou-Alibori University Hospital Centre when it was 85,110 FCFA (129.94 Euro) at the Evangelical Hospital of Bembéréké with extremes of 25,300 (38.63 Euro) and 160,800 FCFA (245.50 Euro). These amounts are far beyond the purchasing power of rural families.

### 3.5. Treatment of SBE

#### 3.5.1. Modern Treatment and Care

Only one-third of the victims seek medical care in the context of the treatment of snake bites in Benin [13]. Indeed, the management of SBE suffers from significant failure in Benin, which could be due to poor health system, organization of emergencies, lack of equipment, and adequate treatment of the first emergency [23]. However, faulty treatment increases the severity of SBE [14]. The latter also reported that first aid (scarification, tourniquet, and incisions) should be reduced to avoid blockage of blood circulation, necrosis, and haemorrhages.

Immunotherapy or antivenom administration remains the only specific effective treatment of SBE [7]. Symptomatic and supportive medical treatment consisting of analgesics, anti-inflammatory drugs, antihaemorrhagics, or surrogate blood clotting factors (whole blood, fresh frozen plasma, and packed red blood cells) is often used when available. Superinfection of affected parts can, if they do not receive adequate care, lead to disability and/or permanent sequelae [14]. Given the very small percentage of SBE victims who attend health facilities in Benin, communities should be educated, sensitized, and sufficiently informed of the benefits of modern medicine to contribute to the reduction in mortality related to snakebites. Thus, through posters and communications in local languages, it will be necessary to inform the population about the risks of SBE.

#### 3.5.2. Availability of Antivenin Sera in Health Facilities in Benin

There is a recognized insufficiency of antivenom sera in Africa, especially in rural health facilities [2, 35]. The prohibitive cost (23,000 CFA francs or 35 euros) and inadequate storage facilities further exacerbate this situation [15, 36]. The availability of antivenom and its use in public health facilities were evaluated [34]. Although antivenom was available in most health facilities, it was not being used following the appropriate protocols due to a lack of training of medical personnel. Later, a clinical trial was carried out using Antivipmyn® Africa (a lyophilized antivenom) in Benin from July 2005 to July 2006 [24]. It was successful, making Antivipmyn® Africa a usable antivenom in Benin. In addition, Inoserp® Pan-Africa, a new antivenom that suits African conditions, has been recognized as effective as other antivenin sera used in Africa (especially FAV Africa, which is no longer available, and Antivipmyn ® Africa) [37].

#### 3.5.3. Traditional Treatment of SBE

In Benin, ophidian envenomation is one of the conditions causing an almost systematic rescue to traditional medicine [11]. Financial constraints and cultural beliefs [13] compel more than 80% of snakebite victims to turn to traditional healers [29]. While it is true that few specific studies have been carried out on the use of plants in the management of SBE in Benin, a recent study (Table 4) [38] emphasized the need for ethnobotanical and pharmacological studies to increase the potential of discovering leading antivenom compounds. *Securidaca longepedunculata, Annona senegalensis,* and *Trichilia emetica* are, for instance, are used to prevent SBE in Benin [47].

**Figure 3:**
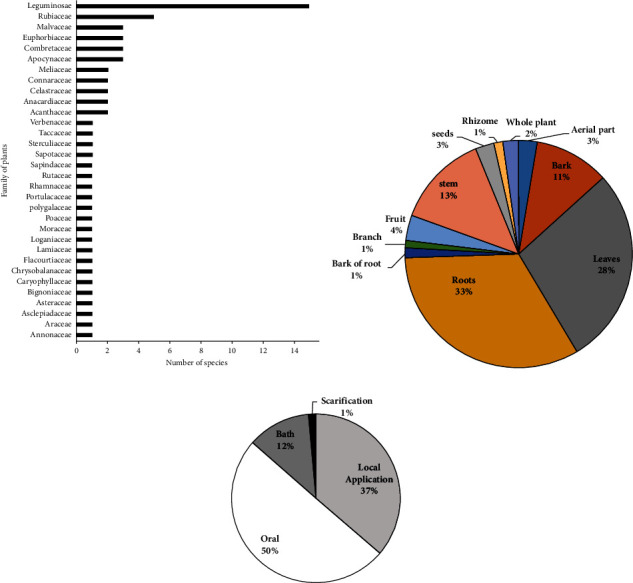
(a) Distribution of plant species used against snakebite in Benin within families; (b) medicinal plant parts used against snakebite in Benin; (c) administration routes used for medicinal plants used against snakebite in Benin.

The most represented families are Leguminosae and Rutaceae, respectively, with 14 and 6 species, followed by Apocynaceae, Connaraceae, Euphorbiaceae, Malvaceae, and Mimosaceae, each represented by 3 species of plants (Figure 3(a)). These families of plants have the inhibiting substances of snake venom. This would justify the fact that they were also mentioned as having antiophidic capacities in other countries including Nigeria, Uganda, Pakistan, Tanzania, Ethiopia, Djibouti, Bangladesh, India, and China [48–53]. In the formulation of antivenoms, roots (33%) and leaves (28%) are parts of plants commonly used by local communities. These remedies are administered much more orally (50%) than by local application (37%) and bath (12%) (Figures 3(b) and 3(c)). Scarification is certainly the least used method of administration to avoid infecting the affected area and creating necrosis.

Originally, people used plants or plant extracts to respond to various health concerns. Rural communities in developing countries, relying on their vast knowledge of the virtues of plants, in seeking therapies to treat SBE, resort to medicinal plants daily [53]. The use of these plants has not yet been approved by a public health organization for reasons of efficacy, dosage, and toxicity. The inventory of plants used by communities in the management of SBE does not necessarily imply their effectiveness but is a step towards finding new active ingredients for the manufacture of new drugs and/or antivenom sera. The issue of the dosage of these plant extracts in their use is also a matter of concern. Note also that some plant species have toxic substances, e.g., the genus *Aristolochia* [54], and therefore, their use must be done with great caution.

## 4. Future Perspectives

SBE is a major public health issue in sub-Saharan Africa. In Benin, SBE has been the subject of studies highlighting the importance of the prevalence of this disease beyond statistical disparities. The incidence and mortality of SBE are underestimated as a result of the inadequate reporting system. Health facilities do not usually have an adequate system for collecting and transmitting data at the central level to facilitate the provision of epidemiological information. What can be done to improve case notifications and data collection in both public and private health facilities? It would be imperative to revitalize the system for reporting SBE in order to have better epidemiological data. The evaluation of the importance, evolution, and severity of the problem at the regional and national levels must be supported by the multiplication of specific studies and the establishment of a specific information system of SBE for the development of a better care strategy. What could be the evolution of the incidence and mortality of SBE in Benin from 2003 to the present day?

Statistics are therefore established based on hospital records from public health facilities, while most victims are treated with traditional medicine. While it is true that the question of financial means has something to do with it, it is also necessary to ask whether the communities are sufficiently informed about the consequences of poor-quality first aid. The development and popularization of an information-education-communication program or strategy or plan for communities is necessary to prevent SBE and improve the management of first aid.

The use and effectiveness of antivenom immunotherapy in the treatment of SBE are well established. However, the question of the accessibility of antivenom is still a big challenge. In addition, the remoteness of health hospital and high cost of antivenoms make them inaccessible to communities. It will therefore act as part of the fight against SBE to subsidize the acquisition and distribution of serums in order to make them more. Therefore, we should consider the local manufacture of serums adapted to the most dangerous snake species living in our country.

In this same perspective, given the enthusiasm of communities for traditional medicine, it would be imperative to develop a program to promote the endogenous knowledge of communities on the use of plants in the management of SBE. Work should be undertaken on the effectiveness of traditional methods, especially plants used in the treatment of SBE, to guarantee the well-being of mainly rural communities in Benin. It could lead to the integration or association of traditional medicine with modern treatment in health facilities.

## 5. Conclusion

SBE constitutes in terms of their frequency and their severity a major health issue in Benin. In addition, due to the disparity in health statistics available, the incidence of SBE remains high in Benin and deserves greater attention. If retrospective studies have made it possible to have data that are no less negligible, prospective studies are sorely lacking thereof higher accuracy and reliability in the ophidian epidemiological data in Benin. The expensive cost of antivenom, the only recognized effective remedy, forces the majority of victims to resort to traditional treatment as a first line. It is essential to conduct experimental studies on the effectiveness of different traditional treatment methods in order to best contribute to the well-being of the Beninese, mostly rural and poor. Alternative pragmatic approaches that can minimize the risks need to be made available to communities so that they can address the issue on their own in a reasonable and sustainable way.

## Figures and Tables

**Table 1:** General characteristics of included publications.

Category	Results
Type of publication	Original article
	Review
	Conference proceedings
	Theses

Type of study	Retrospective study
	Prospective study
	Household survey
	Clinical investigation
	Treatment recommendation

Language	English
	French

Year of publication	1900–2023

**Table 2:** Summary of ad hoc retrospective studies conducted in different areas of Benin.

Place	Year (s)	Incidence	Mortality (%)	Reference
Southern of Benin	1985–1996	25–450	0–2.15	[13]
Borgou Departmental Hospital in Parakou	2002–2004	119	1.68	[15]
Evangelical Hospital of Bembéréké (HEB)	2003	96	5.2	[16]
Borgou Departmental Hospital in Parakou and Evangelical Hospital of Bembéréké (HEB)	2002–2004	49.4	1.69	[17]
Zone hospitals Basslila and Natitingou	2000–2004	38.02	2.75	[18]
Pobè (Plateau-Bénin)	2008	40.2	7.2	[19]
Borgou Departmental Hospital in Parakou	2006–2008	32	37.5	[20]
Zone Hospital Tchaourou	2007–2011	103	5.2	[21]
Zone hospitals of “Ordre de Malte” of Djougou and Bassila	2017-2018	204	2.9	[22]

**Table 3:** Summary of ad hoc prospective studies conducted in different areas of Benin.

Study area	Year (s)	Incidence	Mortality (%)	Reference
Borgou Departmental Hospital in Parakou	February 2007–August 2008	51	13.73	[20]
Hospital “Saint Jean de Dieu” of Tanguita and Hospital of “Ordre de Malte” of Djougou and Bassila	15 June–5 September 2013	100	4	[25]
Borgou Departmental Hospital in Parakou and Evangelical Hospital of Bembéréké (HEB)	January–July 2016	71	7	[26]
Hospital “Saint Jean de Dieu” of Tanguita	31 July–31 October 2019	53	3.77	[27]

**Table 4:** Some plants used in the treatment of SBE in Benin.

Scientific name	Family	Parts used	Mode of application	References
*Lepidagathis anobrya* Nees	Acanthaceae	Roots	O	[38]
*Phaulopsis imbricata* (forssk.) Sweet	Acanthaceae	Whole plant, aerial part	O	[38]
*Lannea acida* A.Rich.	Anacardiaceae	Roots	Ap	[38]
*Annona senegalensis* Pers.	Annonaceae	Roots, leaves, stem	O, Ap	[38, 39]
*Steganotaenia araliacea* Hochst.	Apocynaceae	Roots, leaves, bark	Ap, bath	[38, 40]
*Strophanthus sarmentosus* DC.	Apocynaceae	Leaves, stem	O, bath	[38]
*Thevetia neriifolia* Juss. ex Steud.	Apocynaceae	Roots	Ap, O	[38]
*Amorphophallus johnsonii* N.E.Br.	Araceae	ND	ND	[40]
*Leptadenia hastata* Vatke	Asclepiadaceae	Aerial part	O	[38, 40]
*Acanthospermum hispidum* DC.	Asteraceae	Leaves	Ap	[38]
*Stereospermum kunthianum* Cham.	Bignoniaceae	Bark of roots	Ap, O	[38, 41]
*Afzelia africana* Pers.	Leguminosae	Roots	ND	[39]
*Polycarpaea corymbosa* (L.) Lam.	Caryophyllaceae	ND	ND	[40]
*Maytenus senegalensis* (Lam.) Exell	Celastraceae	Stem	O, bath	[38]
*Gymnosporia senegalensis* (Lam.) Loes.	Celastraceae	Stem, leaves	O, bath	[40]
*Parinari curatellifolia* Planch. ex Benth.	Chrysobalanaceae	Leaves	Bath	[38]
*Combretum hypopilinum* Diels	Combretaceae	Roots	O	[38]
*Byrsocarpus coccineus* Schumach. & Thonn.	Connaraceae	Stem	Ap, O	[38]
*Rourea coccinea* (Schumach. & Thonn.) Benth.	Connaraceae	Stem, leaves	O	[40]
*Tacca leontopetaloides* (L.) Kuntze	Taccaceae	Rhizome, bark, seeds	O	[38]
*Mallotus oppositifolius* (Geiseler) Müll. Arg.	Euphorbiaceae	Leaves	O	[38]
*Manihot esculenta* Crantz	Euphorbiaceae	Branch, leaves, roots	O, Ap	[38]
*Manihot glaziovii* Müll. Arg.	Euphorbiaceae	Leaves, sap	Ap	[38, 40]
*Cassia tora* L.	Leguminosae	Seeds, leaves	O	[38]
*Cassia occidentalis* L.	Leguminosae	Leaves	AP	[38]
*Daniellia oliveri* (Rolfe) Hutch. & Dalziel	Leguminosae	Roots, stem	O, bath	[38, 41]
*Parkia biglobosa* (Jacq.) G.Don	Leguminosae	Bark	ND	[38]
*Piliostigma thonningii* (Schum.) Milne-Redh	Leguminosae	Leaves, roots	O, bath	[38, 41]
Swartzia madagascariensis Desv.	Leguminosae	Leaves	O, bath	[38]
*Tamarindus indica* L.	Leguminosae	Seeds, whole plant	O, Ap	[38, 42]
*Solenostemon monostachyus* (P. Beauv.) Briq.	Lamiaceae	Leaves	O	[38]
Strychnos spinosa Lam.	Loganiaceae	Roots	O	[38]
*Abelmoschus moschatus* Medik.	Malvaceae	ND	ND	[40]
*Hibiscus rostellatus* Guill. & Perr.	Malvaceae	Leaves	ND	[40]
*Sida rhombifolia* L.	Malvaceae	ND	ND	[40]
*Khaya senegalensis* (desv.) A.Juss.	Meliaceae	Roots	ND	[39]
*Trichilia emetica* Vahl	Meliaceae	Roots	O	[39]
*Acacia polyacantha* Willd.	Leguminosae	Bark, roots	O	[38]
*Acacia sieberiana* DC.	Leguminosae	Roots	Ap	[38]
*Entada Africana* Guill. & Perr.	Leguminosae	Roots, stem	Ap, O, bath	[38, 41]
*Prosopis africana* (Guill. & Perr.) Taub.	Leguminosae	Bark	ND	[39]
*Afrormosia laxiflora* (Baker) Harms	Papilionaceae	Stem	Ap	[38]
*Erythrina senegalensis* DC.	Papilionaceae	Bark, leaves	Ap	[38]
*Cymbopogon citratus* (DC.) Stapf	Poaceae	Leaves	Ap	[38, 40]
*Securidaca longepedunculata* Fresen	Polygalaceae	Bark of roots	O, Ap	[38, 39]
*Talinum triangulare* (Jacq.) Willd.	Portulacaceae	ND	ND	[38]
*Ziziphus mauritiana* Lam.	Rhamnaceae	Roots	Ap	[38, 40]
*Chassalia kolly* (Schumach.) Hepper	Rubiaceae	Leaves, roots	Ap, O	[38]
*Citrus limon* (L.) Osbeck	Rutaceae	Fruit	O, Ap	[39]
*Feretia apodanthera* Delile	Rubiaceae	Bark, roots	O	[38, 40]
*Pavetta crassipes* K. Schum	Rubiaceae	Roots, seeds, fruits, leaves	O, scarification	[38, 40]
*Sarcocephalus* latifolius (Sm.) E.A.Bruce	Rubiaceae	Roots	ND	[39]
*Blighia sapida* K.D. Koenig	Sapindaceae	Bark	Ap	[43]
*Vitellaria paradoxa* C.F.Gaertn.	Sapotaceae	Roots, bark	O	[38, 39]
*Sterculia* setigera Delile	Sterculiaceae	Roots	Ap, O	[38]
*Vitex simplicifolia* Oliv.	Verbenaceae	Roots, leaves	O	[38]
*Milicia excelsa* (Welw.) C.C.Berg	Moraceae	ND	ND	[41]
*Combretum collinum* Fresen	Combretaceae	Roots	O	[41]
*Gardenia erubescens* Stapf & Hutch	Rubiaceae	Stem	ND	[41]
*Rourea coccinea* (Schumach. & Thonn.) Benth.	Connaraceae	Leaves, stem	O	[41]
*Haematostaphis barteri* Hook.f.	Anacardiaceae	Leaves, bark, roots	O	[44]
*Grewia lasiodiscus* K.Schum.	Moraceae	Fruit	O	[42]
*Flacourtia indica* (Burm.f.) Merr.	Flacourtiaceae	Leaves, roots	ND	[45]
*Erythrophleum suaveolens* (Guill. & Perr.) Brenan	Leguminosae	ND	ND	[46]

Mode of administration: O: oral application; Ap: local application; ND: not reported.

## Data Availability

All required datasets on which the conclusion of the paper depends on are included within the manuscript.
